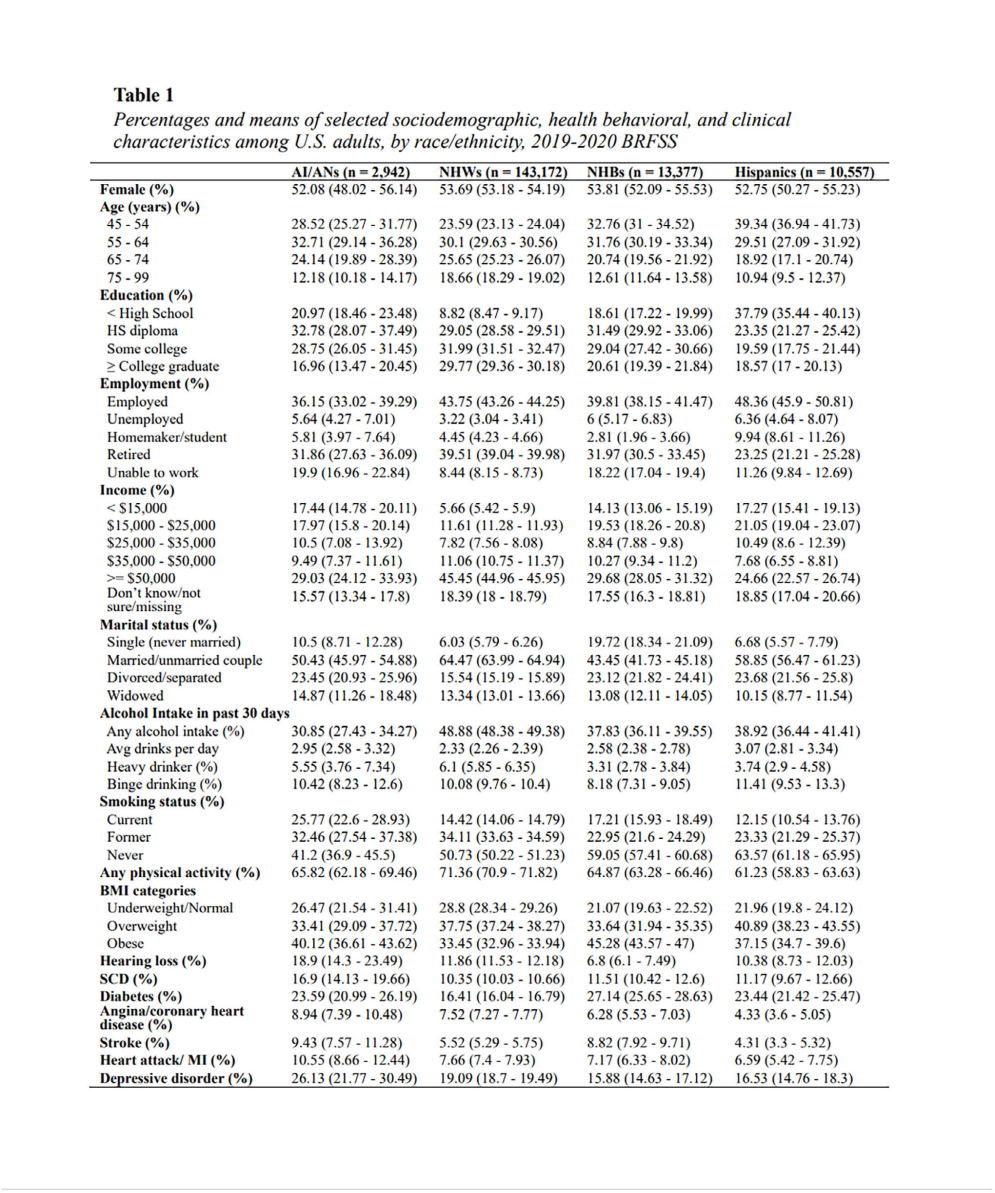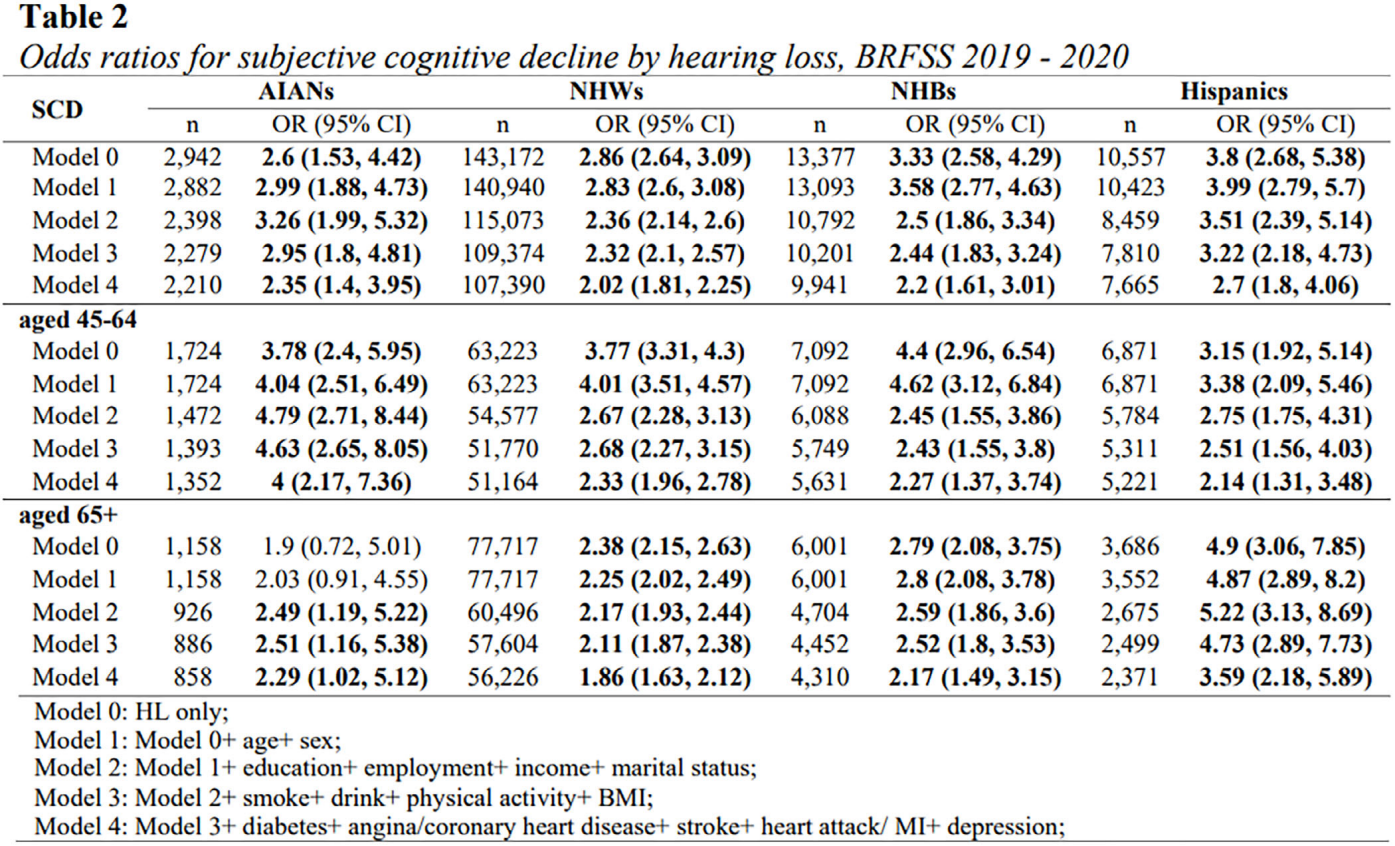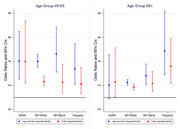# Associations of hearing loss with subjective cognitive decline among American Indians and Alaska Natives and other race/ethnic groups: results from the BRFSS

**DOI:** 10.1002/alz.093052

**Published:** 2025-01-09

**Authors:** Lingling Li, Manxi Yang, Spero Manson, Joan O'Connell, Luohua Jiang

**Affiliations:** ^1^ University of California Irvine, Irvine, CA USA; ^2^ University of Colorado Anschutz Medical Campus, Aurora, CO USA

## Abstract

**Background:**

Prior research has established a relationship between hearing loss (HL) and cognitive decline. However, this association remains unclear in the American Indian/Alaska Native (AI/AN) population. Given the high prevalence of subjective cognitive decline (SCD) and HL in the AI/AN population, exploring this relationship is crucial. This study investigated the HL‐SCD association among AI/AN and other racial/ethnic participants of the Behavioral Risk Factor Surveillance System (BRFSS).

**Method:**

This study analyzed data from 170,048 participants aged 45+ in the 2019‐2020 BRFSS, encompassing AI/AN (n = 2,942), non‐Hispanic White (NHW, n = 143,172), non‐Hispanic Black (NHB, n = 13,377), and Hispanic (n = 10,557) adults. Sociodemographic characteristics, health behaviors, and comorbidities were reported across the four groups. HL was defined as self‐reported deaf or having serious difficulty hearing, and SCD was defined as self‐repoted worsening confusion or memory loss in the past 12 months. Logistic regressions were employed to assess the HL‐SCD association, adjusting for demographic, behavioral, and clinical variables. Additionally, age‐specific analyses were conducted for the 45‐64 and 65+ age cohorts.

**Result:**

AI/ANs exhibited higher prevalence of HL, at 18.92% (95% CI: 14.3% ‐ 23.53%), and SCD, at 16.9% (95% CI: 14.13% ‐ 19.66%), compared to other groups (Table 1). A consistent and significant positive association between HL and SCD was observed across all groups. For AI/AN participants, the fully adjusted odds ratio (OR) for SCD associated with HL was 2.35 (95% CI: 1.4 ‐ 3.95). The ORs for NHW, NHB, and Hispanic participants were 2.02 (95% CI: 1.81 ‐ 2.25), 2.2 (95% CI: 1.61 ‐ 3.01), 2.7 (95% CI: 1.8 ‐ 4.06) respectively (Table 2). The study also revealed that the HL‐SCD association varies by age. Among AI/ANs, NHWs, and NHBs, this association is more pronounced in the 45‐64 age group. Conversely, for Hispanics, it is stronger in the 65+ age group (Figure 1).

**Conclusion:**

Our study found a significant association between HL and SCD in the AI/AN population, consistent with other racial/ethnic groups. We also found age was a key effect modifier in this association. These findings suggest the potential value of routine hearing tests in older AI/AN adults for early dementia detection and prevention.